# Hydrogen peroxide and sodium hypochlorite disinfectants are more effective against *Staphylococcus aureus* and *Pseudomonas aeruginosa* biofilms than quaternary ammonium compounds

**DOI:** 10.1186/s13756-018-0447-5

**Published:** 2018-12-17

**Authors:** Caitlinn B. Lineback, Carine A. Nkemngong, Sophie Tongyu Wu, Xiaobao Li, Peter J. Teska, Haley F. Oliver

**Affiliations:** 1 Department of Food Science, Purdue University, 745 Agriculture Mall Drive, West Lafayette, IN 47907 USA; 2 Diversey Inc., Charlotte, NC 28273 USA

**Keywords:** Disinfectant, Biofilms, Efficacy

## Abstract

**Background:**

Antimicrobial disinfectants are used as primary treatment options against pathogens on surfaces in healthcare facilities to help prevent healthcare associated infections (HAIs). On many surfaces, pathogenic microorganisms exist as biofilms and form an extracellular matrix that protects them from the antimicrobial effects of disinfectants. Disinfectants are used as all-purpose antimicrobials though very few specifically make biofilm efficacy claims. The objective of this study was to evaluate the efficacy of eight registered disinfectants (six registered by the Environmental Protection Agency and two products registered in by the European Chemical Agency) with general bactericidal claims, but currently no biofilm efficacy claims, against *Staphylococcus aureus* ATTC-6538 and *Pseudomonas aeruginosa* ATCC-15442 biofilms. We hypothesized that hydrogen peroxide and sodium hypochlorite disinfectant products would be more effective than quaternary ammonium chlorides.

**Methods:**

This study tested the bactericidal efficacy of eight registered disinfectant products against *S. aureus* ATCC-6538 and *P. aeruginosa* ATCC-15442 grown on glass coupons using a Center for Disease Control (CDC) biofilm reactor and EPA MLB SOP MB-19. Bactericidal efficacy was determined after treating coupons with disinfectants following standard EPA MLB SOP MB-20.

**Results:**

Overall, sodium hypochlorite and hydrogen peroxide disinfectants had significantly higher bactericidal efficacies than quaternary ammonium chloride disinfectants. We also found that all tested disinfectants except for quaternary ammonium chloride disinfectants met and exceeded the EPA standard for bactericidal efficacy against biofilms.

**Conclusion:**

In general, bactericidal efficacy against biofilms differed by active ingredient. The efficacies of sodium hypochlorite and hydrogen peroxide disinfectants did not vary between strains, but there were significant differences between strains treated with quaternary ammonium chloride disinfectants.

## Background

Healthcare associated infections (HAIs) are reported to occur in one out of 25 patients daily on average in the US [1] with over 2 million patients contracting HAIs annually [2]. In the USA, the overall incidence of HAIs is estimated to have increased by 36% in the last two decades [3]. Bacterial biofilms account for 65 and 80% of microbial and chronic infections, respectively [4]. A 2012 study suggested that biofilms may serve as a source of infections by periodically releasing planktonic bacterial cells into the environment [5]. The use of disinfectants is critical to preventing transmission of infectious pathogens from contaminated surfaces and medical equipment to patients [6, 7]. Despite emphasis on surface disinfection, pathogenic microorganisms are routinely isolated from the hospital environment [5, 7].

Within healthcare facilities, *Staphylococcus aureus* and *Pseudomonas aeruginosa* are amongst the most problematic pathogens [8] with *S. aureus* being the second most common pathogen that caused HAIs [9]. These pathogens grow on hard non-porous surfaces such as metal pipes and floor drains [10] and develop an extracellular polymeric matrix that protects the cells from adverse conditions [4, 11]. It has also been shown that the biofilm matrix enhances tolerance to disinfectants by encasing the underlying cells [12, 13] and by limiting diffusion of disinfectants into the biofilm matrix [14]. In fact, the bactericidal efficacy of disinfectants on biofilms is much lower compared to the efficacy of the same disinfectants against planktonic cells [8, 15–17]. The tolerance of biofilms to disinfectants is dependent on disinfectant active, temperature, and the type of surface [13]. Surface roughness, surface humidity, and the availability of nutrients influence the establishment of biofilms on surfaces [18]. Moist surfaces have been shown to be more favorable for biofilm growth even though biofilms have also been reported to grow on dry surfaces [4, 14].

Disinfectants are primary intervention options against pathogenic organisms on surfaces in healthcare facilities [7, 14] and are used as broad-spectrum antimicrobials [19]. Common antimicrobials used for disinfecting surfaces in healthcare facilities include quaternary ammonium compounds, hydrogen peroxide, and chlorine-based products [6, 17]. There are few published studies that investigate the efficacy of disinfectants on bacterial biofilms at label use concentrations. The objective of this study was to evaluate the efficacy of eight registered disinfectants with general bactericidal claims, but no current biofilm efficacy claims, against *S. aureus* ATTC-6538 and *P. aeruginosa* ATCC-15442 biofilms. We hypothesized that accelerated hydrogen peroxide disinfectant products would be more effective than quaternary ammonium compounds and that sodium hypochlorite disinfectants would be the most effective at eliminating biofilms.

## Methods

### Disinfectants and bacteria strains used in this study

This study tested the bactericidal efficacy of eight registered disinfectant products (Table 1) against *S. aureus* ATCC-6538 and *P.s aeruginosa* ATCC-15442. These strains are EPA-defined strains required for biofilm disinfectant efficacy registration claims [20]. Disinfectants were tested at label contact times and concentrations. Phosphate buffered saline (PBS) was used as a control.

**Table 1:** Active ingredients and contact times for disinfectant products tested in this study

Disinfectant Product “Name” (used in the manuscript or figures)^a^	Disinfectant Active Ingredient(s)^c^	Dilution	Active Level at Use^e^	Label Contact Time (mins)^f^
HP1	0.5% hydrogen peroxide	RTU^d^	0.5%	1
HP2	0.5% Hydrogen Peroxide	RTU	0.5%	1
HP3^b^	7.0% hydrogen peroxide	RTU	7.0%	1
HP4^b^	7.2% hydrogen peroxide	1:20	0.36%	5
HP5	4.25% hydrogen peroxide	1:16	0.27%	5
Q1	6.67% octyl decyl ammonium chloride; 2.67% docctyl dimethyl ammonium chloride; 4.00% didecyl dimethyl ammonium chloride; 8.90% alkyl (C_14_, 50%; C_12,_ 40%; C_16,_ 10%) dimethyl benzyl ammonium chloride	1:256	0.087%	3
Q2	8.704% didecyl dimethyl ammonium chloride; 8.190% n-alkyl (C_14_, 50%; C_12,_ 40%; C_16,_ 10%) dimethyl benzyl ammonium chloride	1:256	0.066%	10
SH1	1.312% sodium hypochlorite	RTU	1.312%	4

^a^ Naming scheme abbreviates the active ingredients of the products used in this study and differentiates products with the same class of active ingredients by numbers

^b^ ECHA registered products

^c^ Active ingredient concentration

^d^ Ready to Use

^e^ Active ingredient concentration after dilution

^f^ Defined EPA label contact time in minutes

### Biofilm development on borosilicate glass coupons

Biofilms were grown using EPA Standard Operation Procedure (SOP) MB-19 for biofilms using a Center for Disease Control (CDC) biofilm reactor (Biosurfaces Technologies, Inc., Bozeman, MT). Borosilicate glass coupons (1.27 ± 0.013 cm; Biosurface Tech, Inc.) were used as carriers in the CDC biofilm reactor. The borosilicate glass coupons were placed in rods each containing three coupons. The biofilm reactor was positioned on a hotplate stirrer (Talbays, Thorofare, NJ) and filled with 500 mL of first phase growth media (Table 2). The media was inoculated with 1 mL of bacterial culture greater than or equal to 10^7^ CFU/mL (Table 2). This formed the batch phase. Each of the test microbes began to adhere to the coupons for 24 h under the conditions defined in Table 2. The cells were subsequently grown in a continuous stirred tank reactor (CSTR) growth phase; 20 L of growth media (detailed in Table 2; TSB; Becton, Dickinson and Company, Sparks, MD) was pumped (Cole-parmer, Barrington, IL) through the reactor at a rate of 30 ± 2 min residence time for both *S. aureus* and *P. aeruginosa*.

**Table 2:** Growth conditions for *S. aureus* and *P. aeruginosa* biofilms

Bacteria Strain	Hotplate Stirrer Settings	Test Culture preparation	Batch phase growth medium 24 h	CSTR^a^ growth medium 24 h
*S. aureus* ATCC-6538	60 ± 5 rpm at 36 ± 1 °C	Frozen stock with 10 ml TSB (30 g TSB/L) overnight at 36 ± 1 °C	3 g/L TSB	1 g/L TSB
*P. aeruginosa* ATCC-15442	125 ± 5 rpm at 21 ± 2 °C	Frozen stock with 10 mL TSB (300 mg TSB/L) overnight at 36 ± 1 °C	300 mg/L TSB	100 mg/L TSB

^a^ Continuously stirred tank reactor (CSTR) phase

### Disinfectant efficacy testing

The efficacy of disinfectants against single strain biofilms was determined using EPA MLB SOP MB-20 [20]. Each rod contained three coupons and was rinsed by dipping in dilution water (1.25 mL KH_2_PO_4_ + 5.0 mL MgCl_2_·6H_2_O). The target density for each coupon was 7.5–9.0 CFU/coupon for *S. aureus* and 8.0–9.5 CFU/coupon for *P. aeruginosa* per EPA MLB SOP MB-20. Coupons were placed in a 50 mL sterile conical tube (Corning Science, Mexico) for treatment and enumeration; coupons were individually evaluated. Five biological replicates were conducted for quaternary ammonium compounds due to known high variability [21]. Three biological replicates were conducted for sodium hypochlorite and hydrogen peroxide testing based on previous work conducted by our group [22]. Each biological replicate for all test products was composed of five technical replicates. Three control coupons were used for each test. Disinfectant product (four mL) was added to each sterile conical tube containing a coupon. Coupons were dipped in dilution water prior to transferred into the tube to remove planktonic cells. Disinfectants were left in contact with the coupons for the label contact times at room temperature (Table 1). Four mL of PBS was added to control coupons. Disinfectant products were neutralized at the label-defined contact time with 36 mL neutralizing buffer solution (1 L H_2_O + 5.2 g Difco neutralizing buffer; Becton, Dickinson and Company Sparks, MD). The treated coupons underwent a rotational series of vortexing (30 s) and sonication using an ultra-sonic water bath (Cole-Parmer Instrument Company, Chicago, IL) at 45 Khz for 30 s three times to release the biofilms from the coupons and suspend the bacteria in solution [20].

The control samples were quantified by serial dilution and spread plating on Tryptic Soy Agar (TSA; BD Biosciences, San Jose, CA) for *S. aureus* and Reasoner’s 2a Agar (R2a; Becton, Dickinson and Company Sparks, MD) for *P. aeruginosa* following EPA MLB SOP MB-20 [20]*.* Coupons treated with quaternary ammonium chloride disinfectants were serially diluted and plated due to high cell recovery; coupons treated with hydrogen peroxide and sodium hypochlorite-based disinfectants were not serially diluted. Ten mL aliquots from each diluted sample were vacuum-filtered onto a membrane filter (0.2 μm pore; Pall Corporation, Port Washington, NY). Membrane filters were plated onto TSA and R2a agar for *S. aureus* and *P. aeruginosa,* respectively, and incubated at 37 °C for 48 ± 4 h prior to estimation.

### Statistical analyses

All statistical analyses were performed using SAS 9.4 (SAS Institute, Cary, NC). CFU log_10_ reductions were calculated and normalized relative to the number of CFUs on control coupons. Disinfectant products were grouped based on the main active ingredients: sodium hypochlorite (1 product), hydrogen peroxide (5 products), and quaternary ammonium compounds (2 products). The data were fitted in a generalized linear mixed model with Proc Glimmix procedure to determine if there were significant differences in log_10_ reductions among disinfectants both by active category and product (*n* = 56; α = 0.05). Least Squares Means with Tukey’s adjustment were used to elucidate the trend of the identified significant differences.

## Results

### Hydrogen peroxide- and sodium hypochlorite-based disinfectant products had similar bactericidal effects against both *S. aureus* and *P. aeruginosa* biofilms

**Fig. 1:**
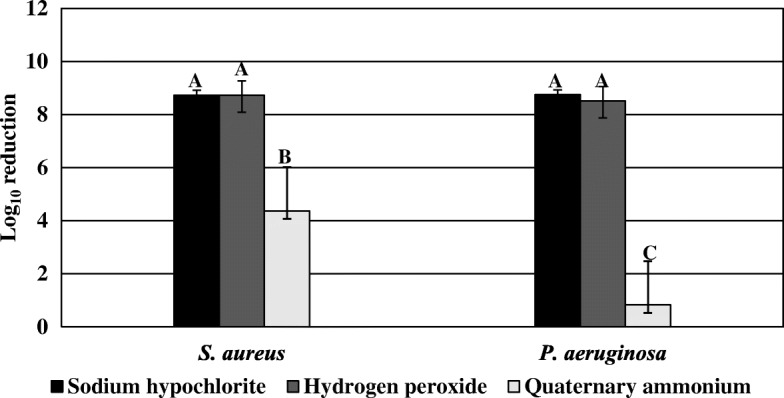
Comparison of active ingredient class by strain

**Fig. 2:**
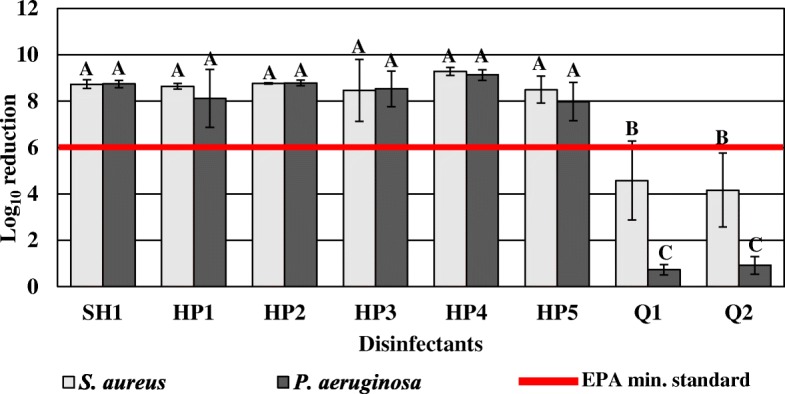
Comparison of EPA registered disinfectants by strain

Regardless of bacterial strain, hydrogen peroxide and sodium hypochlorite disinfectants achieved a greater overall bactericidal efficacy than quaternary ammonium disinfectants, both by active ingredient category (*P* < 0.0001) (Fig. 1) and by individual product (*P* < 0.0001) (Fig. 2). Overall, *S. aureus* biofilms had a greater overall log reduction than *P. aeruginosa* biofilms after disinfection regardless of active ingredient category (*P* < 0.0001; Fig. 1) or the specific product applied (*P* = 0.0002; Fig. 2). A comparison of disinfectants by active ingredient category showed a significantly higher log reduction of *S. aureus* biofilms (4.37 log reduction) than *P. aeruginosa* biofilms (0.82 log reduction) by quaternary ammonium products (*P* < 0.0001; Fig. 1). Coupons disinfected with quaternary ammonium chloride products had on average 4.75 ± 1.69 *S. aureus* CFU/coupon (4.37 log reduction) and 8.02 ± 0.60 *P. aeruginosa* CFU/coupon (0.82 log reduction) post-treatment. There were no significant differences in bactericidal efficacy against *S. aureus* and *P. aeruginosa* biofilms after disinfection by hydrogen peroxide or sodium hypochlorite products. *S. aureus* and *P. aeruginosa* biofilms had an average log density of 0.33 ± 0.06 CFU/coupon (8.73 log reduction) and 0.30 CFU/coupon (8.51 log reduction) after disinfection with hydrogen peroxide disinfectants, respectively (Fig. 1) per EPA MLB SOP MB-20, 0.30 CFU/coupon is the reported detection limit when no cells are recovered thus there is no calculable standard deviation. *S. aureus* and *P. aeruginosa* coupons disinfected with the sodium hypochlorite product had mean log densities of 0.30 CFU/coupon (8.73 log reduction) and 0.33 ± 0.08 CFU/coupon (8.75 log reduction), respectively (Fig. 1).

When evaluating each disinfectant individually, both quaternary ammonium products exhibited significant differences in bactericidal efficacy against *S. aureus* and *P. aeruginosa* biofilms (Fig. 2). Specifically, *S. aureus* biofilms were significantly more reduced than the *P. aeruginosa* biofilms when treated with Q1 (*P* < 0.0001) and Q2 (*P* = 0.0001) (Fig. 2). No other significant differences were observed between *S. aureus* and *P. aeruginosa* biofilms disinfected by hydrogen peroxide or sodium hypochlorite products.

### Hydrogen peroxide and sodium hypochlorite disinfectants had significantly higher bactericidal efficacy against *S. aureus* biofilms than quaternary ammonium products

There were significant differences in bactericidal efficacy among tested disinfectants against *S. aureus* both by active ingredient category (*P* < 0.0001; Fig. 1) and by individual product (*P* < 0.0001; Fig. 2). Products with hydrogen peroxide and sodium hypochlorite as active ingredients achieved significantly higher *S. aureus* log reduction than quaternary ammonium-based products (*P* < 0.0001; (Fig. 1). Specifically, sodium hypochlorite disinfectant SH1 and all hydrogen peroxide disinfectants (HP1, HP2, HP3, HP4, and HP5) individually by product were more effective against *S. aureus* biofilms than either of the two tested quaternary ammonium products (*P* < 0.0001; Fig. 2). There was no significant difference in bactericidal efficacy against *S. aureus* biofilms treated with Q1 compared to Q2 (*P* > 0.05; Fig. 2). There were no significant differences in disinfection performance among the aforementioned hydrogen peroxide and sodium hypochlorite products collectively (*P* > 0.05; Fig. 2).

### Hydrogen peroxide and sodium hypochlorite disinfectants were more bactericidal against *P. aeruginosa* biofilms compared to quaternary ammonium compounds

Bactericidal efficacy was significantly different among disinfectants applied to *P. aeruginosa* biofilms both by active ingredient category (*P* < 0.0001; Fig. 1) and by specific product (*P* < 0.0001; Fig. 2). Hydrogen peroxide and sodium hypochlorite-based disinfectants were more effective against *P. aeruginosa* biofilms than quaternary ammonium products (*P* < 0.0001; Fig. 1). Specifically, sodium hypochlorite disinfectant (SH1) and all hydrogen peroxide disinfectants (HP1, HP2, HP3, HP4, and HP5) by product individually achieved significantly higher bactericidal efficacy against *P. aeruginosa* than either of the quaternary ammonium chloride products Q1 and Q2 (*P* < 0.0001; Fig. 2). There were no significant differences among hydrogen peroxide products or between sodium hypochlorite disinfectant and hydrogen peroxide products (*P* > 0.05). There was no statistically significant difference in efficacy between the two quaternary ammonium products (*P* > 0.05).

## Discussion

In this study, we tested eight registered disinfectants under label use conditions against *S. aureus* and *P. aeruginosa* biofilms using EPA methods MB-19 and MB-20. We found statistically significant quantitative differences among disinfectant active ingredients and products against *S. aureus* and *P. aeruginosa.* Specifically, we found (i) statistically significant differences in disinfectant efficacy among disinfectants, (ii) similar performance of hydrogen peroxide and sodium hypochlorite-based products against *S. aureus* and *P. aeruginosa* biofilms, and iii) significantly higher bactericidal efficacy of quaternary ammonium-based products against *S. aureus* than *P. aeruginosa*. Bacterial biofilms are common on a wide range of surfaces made of different materials and have been reported to be present in drains, metal pipes [10], sanitizing bottles, trolleys and clipboards [23] thus are potential sources of HAIs.

### Disinfectant efficacy varies by active ingredient

We found significant differences among quaternary ammonium compound disinfectants compared to hydrogen peroxide and sodium hypochlorite disinfectants. The quaternary ammonium compounds did not achieve the current EPA regulation minimum stating that the disinfectant must decrease the bacterial load by 10^6^ CFU [24]. The findings in this study underscoring low quaternary ammonium compound efficacy against laboratory-grown biofilms. This raises concerns for healthcare facilities as quaternary ammonium disinfectants are reported to be among the most commonly used disinfectants in healthcare facilities [25, 26]. Quaternary ammonium compounds are cationic in nature [27, 28] and their interaction with a negatively charged biofilm matrix could inhibit their bactericidal efficacy [29]. Tseng et al. found that the efficacy of tobramycin, a positively charged antibiotic, was decreased as it was sequestered at the surface of the negatively charged biofilm matrix thus did not penetrate the matrix to contact underlying viable *P. aeruginosa* cells [29]. In addition, the bactericidal efficacy of quartenary ammonium compounds may fluctuate because they have been shown to be biogradeble under aerobic condictions [30].

Hydrogen peroxide and sodium hypochlorite disinfectants were effective against *P. aeruginosa* and *S. aureus* biofilms at the EPA required reduction levels. Hydrogen peroxide and sodium hypochlorite disinfectants have been reported to destroy both the biofilm matrix and the bacteria cells within, making them better anti-biofilm agents [31, 32]. Specifically, sodium hypochlorite disinfectant products irreversibly kill bacterial cells in biofilms by denaturing proteins in the biofilm matrix and inhibiting major enzymatic functions in bacterial cells. Although sodium hypochlorite disinfectants at concentrations as low as 0.0219% are effective against the formation of *S. aureus* biofilms [33], the use of sub-lethal concentrations of some sodium containing disinfectants could actually promote the formation of biofilms on environmental surfaces [34]. In a study conducted by West et al. [22], hydrogen peroxide products and sodium hypochlorite products were more effective against both *S. aureus* and *P. aeruginosa* planktonic cells compared to quaternary ammonium. On another note, surfaces disinfected with hydrogen peroxide based antimicrobials have demonstrated significantly lower chances of bacterial regrowth than those disinfected with quaternary ammonium compounds [35]. To this effect, the study by Boyce et al. [35] concluded that the risk of the incidence of HAIs was lower with hydrogen peroxide disinfectants than with the use of quaternary ammonium compounds. Our data suggest that hydrogen peroxide or sodium hypochlorite products should be used in healthcare facilities for routine use, particularly on surfaces prone to biofilm development. However, hydrogen peroxide disinfectants have also been reported to be corrosive on medical equipment such as flexible endoscopes [36] and can discolor metal finishes [37]. Despite these limitations, Alfa et al. [38] also demonstratated that a 0.5% hydrogen peroxide antimicrobial is highly efficient at disinfecting medical devices. Moreover, hydrogen peroxide disinfectants are neither irritating or malodorous [37].

The ability of biofilm matrices to prevent contact between disinfectant products and bacterial cells is complex [39]. Biofilms are characterized by high cell population densities that supply large amounts of polymeric substances, which consequently enables the formation of well-structured, functional matrices [39]. Moreover, biofilm cells are genetically primed to better tolerate disinfectant products compared to plaktonic cells [39, 40]. These features prevent the diffusion of disinfectants and limit bactericidal efficacy [41]. While our study emphasized the efficacy of disinfectants at label concentration and contact time, it did not investigate the efficacy of disinfectants at off label use or with varying environmental effects. Monoculture biofilms will be rare in healthcare environments and soil levels and surface type will vary. Further, this work was conducted on glass coupons per the EPA protocol, which does not necessarily represent how cells will grow on other surfaces (e.g. hard plastics, stainless steel). Recognizing these limitations, more work is needed to investigate other variables that can impact disinfectant efficacy (e.g. dry biofilms) as well as applications in healthcare settings.

## Conclusion

We found that hydrogen peroxide and sodium hypochlorite products are effective against *S. aureus* and *P. aeruginosa* biofilms, which can be common in healthcare facilities. However, quaternary ammonium chloride compounds are not as effective against *S. aureus* and *P. aeruginosa* biofilms grow on hard non-porous surfaces and did not achieve a minimum 6 log_10_ CFU reduction. While further research is warranted to evaluate more complex biofilms in hospital environements, test the efficacy of disinfectants against dry biofilms, and to optimize the bactericidal effects of a combination of different ready to use antimicrobials, infection preventionists should consider the use of hydrogen peroxide and sodium hypochlorite products on surfaces at risk of biofilm development to prevent HAIs.

## Abbreviations

ATCC: American type Culture collection
CFU: Colony forming unit
EPA: Environmental Protection Agency
HAI: Healthcare-associated infection
HP1: 0.5% hydrogen peroxide
HP2: 0.5% hydrogen peroxide
HP3: 7.0% hydrogen peroxide
HP4: 7.2% hydrogen peroxide
HP5: 4.25% hydrogen peroxide
PBS: Phosphate buffered saline
Q1: 22.24% quaternary ammonium compounds
Q2: 16.89% quaternary ammonium
RTU: Ready-to-use
SH1: 1.312% sodium hypochlorite sodium hypochlorite
TSB: Tryptic soy broth
